# Distinct Organotypic Platforms Modulate Rainbow Trout (*Oncorhynchus mykiss*) Intestinal Cell Differentiation In Vitro

**DOI:** 10.3390/cells12141843

**Published:** 2023-07-13

**Authors:** Nicole Verdile, Federica Camin, Radmila Pavlovic, Rolando Pasquariello, Milda Stuknytė, Ivano De Noni, Tiziana A. L. Brevini, Fulvio Gandolfi

**Affiliations:** 1 Department of Agricultural and Environmental Sciences, University of Milan, 20133 Milan, Italy; federica.camin@unimi.it (F.C.);; 2 Department of Veterinary Medicine and Animal Sciences, University of Milan, 26900 Lodi, Italy; radmila.pavlovic1@unimi.it (R.P.); tiziana.brevini@unimi.it (T.A.L.B.); 3 Proteomics and Metabolomics Facility, IRCCS San Raffaele Scientific Institute, 20132 Milan, Italy; 4 Unitech COSPECT—University Technological Platform, University of Milan, 20133 Milan, Italy; milda.stuknyte@unimi.it; 5 Department of Food, Environmental and Nutritional Sciences, University of Milan, 20133 Milan, Italy; ivano.denoni@unimi.it

**Keywords:** aquaculture, sustainable feed, rainbow trout, in vitro intestine, intestinal cells, organotypic culture, organoids, cell differentiation, enterocytes

## Abstract

In vitro organotypic cell-based intestinal platforms, able to faithfully recapitulate the complex functions of the organ in vivo, would be a great support to search for more sustainable feed ingredients in aquaculture. We previously demonstrated that proliferation or differentiation of rainbow trout intestinal cell lines is dictated by the culture environment. The aim of the present work was to develop a culture platform that can efficiently promote cell differentiation into mature enterocytes. We compared four options, seeding the RTpiMI cell line derived from the proximal intestine on (1) polyethylene terephthalate (PET) culture inserts ThinCert™ (TC), (2) TC coated with the solubilized basement membrane matrix Matrigel^®^ (MM), (3) TC with the rainbow trout fibroblast cell line RTskin01 embedded within the Matrigel^®^ matrix (MMfb), or (4) the highly porous polystyrene scaffold Alvetex^®^ populated with the abovementioned fibroblast cell line (AV). We evaluated the presence of columnar cells with a clear polarization of brush border enzymes, the formation of an efficient barrier with a significant increase in transepithelial electrical resistance (TEER), and its ability to prevent the paracellular flux of large molecules but allow the transit of small compounds (proline and glucose) from the apical to the basolateral compartment. All parameters improved moving from the simplest (TC) through the more complex platforms. The presence of fibroblasts was particularly effective in enhancing epithelial cell differentiation within the AV platform recreating more closely the complexity of the intestinal mucosa, including the presence of extracellular vesicles between fibroblasts and epithelial cells.

## 1. Introduction

Aquaculture is continuously searching for more sustainable ingredients to reduce the environmental impact of new feed formulations. The search for appropriate and efficient alternatives is time-consuming and expensive, and it requires the sacrifice of many animals, because extensive feeding trials are essential for assessing the effect of raw materials on the gastrointestinal tract. Cell-based models could represent a valuable support to in vivo experiments. They offer several advantages including direct observation, continuous analysis, limited variability, and reduced use of experimental animals [1]. However, in standard tissue-culture conditions, cell lines proliferate incessantly and lack a completely mature phenotype. Moreover, conventional cell culture settings prevent the physiological interactions both between cell types and with the extracellular matrix, thereby altering cell behavior, specific gene and protein expression, and responsiveness to external stimuli [2,3,4,5]. For these reasons, conventional culture procedures limit the capacity of in vitro systems to generate predictive data and to correlate them with in vivo experiments.

The use of culture inserts makes it possible to reproduce in vitro the mechanisms of nutrient transport across the intestinal epithelium. These inserts create dual-chamber devices where the compartments are physically separated by a porous membrane on which cells grow as a monolayer establishing an efficient barrier. The upper chamber simulates the intestinal lumen, and the cells growing on the membrane mimic the epithelial surface of the intestinal wall [1], whereas the lower compartment represents the vascular system [6].

Recently, we established two epithelial intestinal cell lines (RTpiMI and RTdiMi) from the proximal and distal tracts that characterize the rainbow trout intestine, enriching the availability of RT in vitro model [7]. Interestingly, they shared several similarities with the in vivo intestinal mucosa [8,9,10] being constituted by a heterogeneous cell population of stem cells, transit-amplifying cells, and partially differentiated cells. When these cells are grown in a tissue culture flask, it is possible to regularly propagate them. At present, they have been passaged for up to 50 times without any obvious sign of senescence or morphological alterations.

We previously demonstrated that these cells promptly respond to simple differentiating stimuli, such as increased seeding density or the presence of a cell culture insert, switching from stem cells or transit-amplifying cells toward a more differentiated phenotype [7]. However, since only epithelial cells were present, the model failed to reproduce the complexity of the gut mucosa that, in vivo, is made of a columnar epithelial monolayer layered upon a loose connective tissue, known as the lamina propria. The two are separated by a thin, dense layer of extracellular matrix called the basement membrane, jointly produced by the epithelial cells and the fibroblasts of the laminal propria. Fibroblasts also produce the abundant extracellular matrix that characterizes the connective tissue of the lamina propria, whose main constituents are fibrous proteins such as collagen type I and type IV, elastin, laminin, and glycosaminoglycans [11,12,13]. This layer not only provides the mechanical support to the overlying epithelium, but also actively participates in its proliferation and differentiation through the secretion of growth and signaling factors [14]. In a recent detailed characterization of the RT intestinal stem-cell niche organization, we highlighted the active functional implication of the subepithelial fibroblasts in the regulation of stem-cell proliferation and differentiation, and more generally, of the intestinal homeostasis [9,10]. Indeed, recent data demonstrated that increasingly complex gut in vitro models that closely resemble the native organ have a better predictive power, becoming reliable devices to explore the uptake mechanisms of molecules and compounds across the intestinal epithelial barrier [15,16]. Therefore, we hypothesized that moving the RT epithelial cells from the simple culture environment of the tissue culture flasks to an enriched environment would stimulate their differentiation. While several efforts have been dedicated to the development of advanced intestinal models that recreate the mouse and human epithelial–mesenchymal interface in vitro [14,17,18], only limited work has been performed in fish species [19].

In order to characterize the role of the culture environment, we compared the performances of increasingly complex organotypic cell-based culture systems. Starting with a simple PET culture insert (TC) as a control, we precoated its surface with a thin layer of solubilized murine basement membrane matrix Matrigel^®^ to promote epithelial cell adhesion and differentiation (MM). These two platforms, composed only by epithelial cells, were compared with two others that were enriched with a connective tissue component in two ways. In the first one, RT fibroblasts were embedded within the Matrigel^®^ layer (MMfb); in the second, we seeded the fibroblasts within the synthetic scaffold Alvetex™ (AV). In both cases, intestinal epithelial cells were seeded on top of the fibroblast containing layers.

The aim of this study was to determine how the four platforms influence the morphological and functional differentiation of the intestinal cells. We selected the RTpiMI cell line, derived from the proximal intestine, as this is the functionally more relevant tract since more than 70% of nutrient absorption occurs therein [20].

## 2. Materials and Methods

### 2.1. Cell Lines

We used the RTpiMI cell line, previously established from the proximal intestinal tract [7] and a rainbow trout fibroblast cell line (RTskin01) derived from the dermis, in our laboratory. In both cases, organs were isolated from animals destined to human consumption and, therefore, were not considered as animal experimentation under Directive 2010/63/EU of the European Parliament.

RTpiMI intestinal cells were maintained in complete medium composed of Leibovitz’s culture medium (L-15, Thermo Fisher Scientific, Waltham, MA, USA, cat. no. 11415064) supplemented with 2 mM L-glutamine, 10,000 units/mL penicillin, 10.0 mg/mL streptomycin, 25.0 µg/mL amphotericin B (Merck, Darmstadt, Germany, cat. no. A5955), and 5% fetal bovine serum (FBS, Thermo Fisher Scientific Waltham, MA, USA cat. no. 10270106), indicated as L-15/C_5_. RTskin01 fibroblasts were propagated in the same medium but supplemented with 10% FBS, indicated as L-15/C_10_.

Both cell types were cultured in 75 cm^2^ tissue culture flasks (T75, Sarstedt, Nurmbrecht, Germany, cat. no. 83.3911) and passaged at a 1:3 ratio, when reaching 80% confluency. RTpiMI cells at passages between 30 and 35 and RTskin01 fibroblasts at passages between 20 and 40 were used for the experiments. All cell lines were grown at 20 °C in air, and the medium was replaced twice a week.

### 2.2. Organotypic Cell-Based Platforms

**Figure 1:**
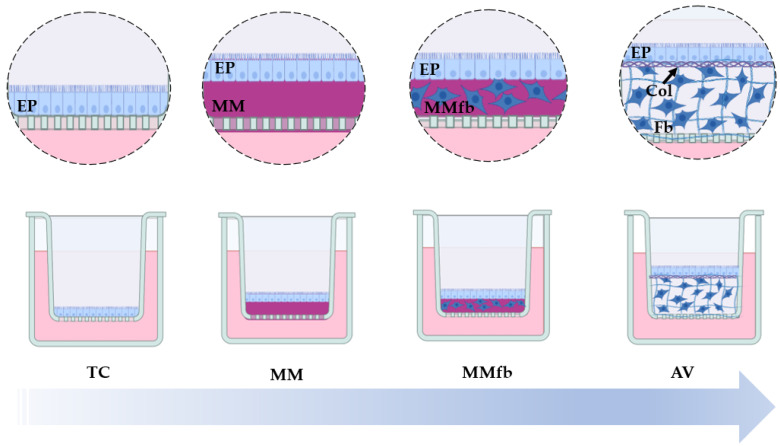
Schematic representation showing the increasingly complex (from left to right) RT gut in vitro platforms used and developed in the present study. RTpiMI epithelial (EP) cells cultured on (1) ThinCert™ culture inserts (TC), (2) TC inserts coated with Matrigel^®^ matrix (MM), (3) TC inserts coated with the Matrigel^®^ matrix enriched with RTskin01 fibroblasts (MMFb), or (4) the synthetic, highly porous Alvetex™ scaffold (AV) populated with fibroblasts (Fb) that synthesize and deposit collagen (Col) between them and at the interface with the apical surface, creating an anchorage site for the epithelial cells (EP). The image was created using Biorender.com.

A scheme of the four culture platforms is presented in Figure 1.

#### 2.2.1. Cell Culture Inserts (TC)

RTpiMI cells were seeded at the final density of 2.5 × 10^5^ cells/cm^2^, onto ThinCert™ polyethylene terephthalate (PET) translucent filter supports with 113 mm^2^ surface and 0.4 µm pore size (Greiner Bio-One, Kremsmunster, Austria, cat. no. 665640).

#### 2.2.2. Cell Culture Inserts Coated with Solubilized Basement Membrane (MM)

ThinCert™ cell culture translucent inserts with the same surface area but with 3 µm pores (Greiner Bio-One, Kremsmunster, Austria, cat. no. 665631) were coated with 125 µL of the solubilized basement membrane matrix Matrigel^®^ (Corning, Corning, NY, USA, cat. no. CLS354234) diluted 1:8 in serum-free L-15 medium. Inserts were incubated at 20 °C for 1 h to allow matrix polymerization. L-15 medium supplemented with 5% FBS was added in both apical (AP) and basolateral (BL) compartments. On the subsequent day, 2.5 × 10^5^ RTpiMI cells/cm^2^ were seeded on the apical surface.

#### 2.2.3. Cell Culture Inserts Coated with Fibroblasts Embedded in Solubilized Basement Membrane (MMfb)

In this platform, 4 × 10^5^ RTskin01 fibroblasts were resuspended in 125 µL of a Matrigel^®^ solution diluted 1:8 in serum-free L-15 medium and layered upon the ThinCert inserts with 3 µm pores (Greiner Bio-One, Kremsmunster, Austria, cat. no. 665631), the same used in the MM platform. After 1 h incubation at 20 °C for allowing polymerization, L-15 medium, supplemented with 10% FCS, was added in both apical (AP) and basolateral (BL) compartments. After 1 week, 2.5 × 10^5^ RTpiMI cells/cm^2^ were seeded on the apical surface.

#### 2.2.4. Alvetex™ Cell Culture Inserts with Embedded Fibroblasts (AV)

RTpiMI cells were seeded on top of Alvetex™ inserts (Reprocell, Orlando, FL, USA) previously embedded with RTskin01 fibroblasts. This inert 3D support consisted of a highly porous, 200 μm thick polystyrene membrane, specifically designed to allow infiltration and free interactions between neighboring cells. Firstly, 12-well AV inserts (cat. no. AVP005-12) were made hydrophilic by submersion in 70% ethanol for 30 min to allow cellular infiltration inside the porous membrane. Subsequently, ethanol was removed with PBS washes, and 75 µL of L15 medium containing 1 × 10^6^ RTskin01 fibroblasts was dispensed onto each AV insert and left in the incubator (20 °C) for 1 h. Subsequently, 3 mL of L-15 medium with 10% FBS was added to the well. RTskin01 fibroblasts were cultured for 28 days, and the medium was replaced twice a week. Thereafter, 75 µL of L-15 medium containing 9 × 10^5^ RTpiMI cells was layered on the apical surface of each well and incubated for 1 h at 20 °C, before adding 3 mL of L-15 complete medium supplemented with 10% FBS.

### 2.3. Sample Processing and General Morphological Evaluation

Samples for morphological analysis were fixed in 4% paraformaldehyde (PFA) in PBS overnight at 4 °C, dehydrated using a graded scale of alcohols, cleared in Histoclear (Histo-Line Laboratories, Pantigliate, Italy, cat. no. R0050CITRO), and embedded in paraffin. Then, 5 µm sections were cut using a rotary microtome (Microm HM335E, Histo-Line Laboratories, Pantigliate, Italy), dehydrated, and stained with hematoxylin and eosin (HE) or 4′,6-diamidino-2-phenylindole (DAPI).

Additionally, to verify whether fibroblast cell number increased with cell culture time, RTski01 were seeded directly on plastic surface or embedded in Matrigel^®^ matrix fixed in 4% PFA in PBS for 30 min, stained with 4′,6-diamidino-2-phenylindole (DAPI), and counted. Given the difference among the platforms, and since it is not possible to count cells in the AV from above, counting was performed on paraffin-embedded sections stained with DAPI. For the same reason, different timepoints were selected for the different platforms. For the plastic surface and MMfb, timepoints 0, 1, and 2 correspond to immediately, 48 h, and 72 h after seeding, respectively. For AV scaffolding, these timepoints correspond to 7, 14, and 28 days of culture. In all cases, five random fields of vision were selected, and all the visible nuclei were counted using the open-source software ImageJ (version 1.53) [21]. Moreover, to evaluate fibroblast distribution within the AV platform, additional paraffin-embedded sections were stained for F-actin. Briefly, sections were brought to distilled water and incubated for 1 h at room temperature with phalloidin-iFluor 594 reagent (Abcam, Cambridge, UK cat. no. AB176757) diluted 1:1000 in PBS. Subsequently, nuclei were counterstained with DAPI.

### 2.4. Immunohistochemistry

Immunohistochemistry analyses on paraffin-embedded sections were used to detect specific targets. In particular, collagen secretion and deposition were analyzed through the immunohistochemical detection of collagen 1 alpha 1 (Col1a1), tight junction formation was studied through the presence of zonula occludens 1 (ZO-1), cellular polarization was analyzed through the detection of sodium–glucose transporter-1 (Sglt1), and actively proliferating cells were detected using proliferating cell nuclear antigen (Pcna). Briefly, slides were brought to boiling in 10 mM sodium citrate buffer, 0.05% Tween-20 (pH 6) in a pressure cooker for 1 min for antigen retrieval. Then, nonspecific binding was prevented by incubating sections in 10% goat serum in PBS (for Col1a1, Pcna or Sglt-1) or 5% bovine serum albumin (BSA) and 0.3% Triton X-100 in PBS (for ZO-1) for 30 min at room temperature. Afterward, samples were incubated with anti-collagen type I antibody (antibodies-online, Aachen, Germany), with FITC-conjugated anti-Zo-1 antibody (Life Technologies, Carlsbad, CA, USA), with anti-Sglt1 rabbit polyclonal antibody (Merck, Darmstadt, Germany, cat. no. 07-1417, 1:200 dilution), or with anti-PCNA mouse monoclonal antibody (Merck, Darmstadt, Germany, cat. no. MAB424, 1:1200 dilution) diluted in 4% bovine serum albumin (BSA) in PBS for 1 h at room temperature. Subsequently, sections were exposed to the adequate Alexa Fluor^TM^ 594 goat anti-rabbit or anti-mouse secondary antibody diluted 1:250 in PBS for 30 min at room temperature. Nuclei were counterstained with DAPI. Secondary antibody controls were performed following the same staining protocol but omitting the primary antibody. The main antibody features are summarized in Table 1.

To evaluate collagen deposition, Col1a1 immunostaining was also performed on RTskin01 fibroblasts cultured on standard plastic surface at the final density of 2.5 × 10^5^ cells/cm^2^ as previously described [7]. In addition, to verify whether collagen deposition increases in parallel with the cell culture time, Col1a1 fluorescence intensity/cm^2^ was quantified using ImageJ [21] on five images randomly selected in each section.

Immunostaining for Col1a1 and ZO-1 was also performed directly on the inserts without paraffin embedding. These samples were analyzed using a FRET-FLIM inverted confocal microscope equipped with the NIS Elements D software, version 5.20. This allowed us to obtain a top view of the 3D platforms and verify the respective distribution of RTski01 and RTpiMI cells.

Rainbow trout intestine sections were prepared from archival material used in previous experiments [10].

### 2.5. Transepithelial Electrical Resistance (TEER)

After epithelial cells were seeded, TEER was measured once a week (day 7, day 14, day 21, and day 28) to monitor epithelial growth and verify the establishment of a functional epithelial barrier in each platform. Briefly, culture medium was replaced with fresh L-15 complete medium. Resistance was measured at three equidistant points of the insert perimeter, and the average was calculated. A blank insert, without cells, was run as a control, and the TEER value was calculated using the following formula:

$$TEER\;value\left(\Omega \cdot {cm}^{2}\right)=\left(Insert\;value-Blank\;insert\;value\right)\times Insert\;surface\;area$$

### 2.6. Epithelial Barrier Efficiency

#### 2.6.1. Paracellular Flux of Large Molecules

To evaluate the efficiency of the epithelial barrier to prevent the paracellular flux of large molecules, we calculated the apparent permeability (P_app_) of 4 kDa fluorescein isothiocyanate–dextran, (FITC–dextran, Merck, Darmstadt, Germany, cat. no. FD4). It was diluted in the culture medium to a final concentration of 100 µg/mL and added to the apical chamber of each platform. After 1, 15, 30, 60, and 120 min, 100 µL of solution was collected from the basolateral compartment and transferred to a black, flat-bottom, 96-well plate (Invitrogen, Waltham, MA, USA, cat. no. M33089). Serial dilutions of the 100 µg/mL 4 kDa FITC–dextran (100 µg/mL, 50 µg/mL, 25 µg/mL, 12.5 µg/mL, 6.25 µg/mL, 3.125 µg/mL, 1.5625 µg/mL, and 0 µg/mL) were prepared as a standard curve. Controls were performed using inserts without cells.

The fluorescence emitted by standards and samples was read using a Synergy HT (BioTek) fluorimeter (em/ex 485/528). The standard curve was used to transform the outcomes of the fluorimeter into the corresponding dextran quantity.

Lastly, to calculate the apparent permeability, the following formula was applied:

(1) $$P_{app}\left(cm\cdot s\right)=\frac{∆Q}{∆t}\times \frac{1}{C_{0}\times A},$$

where ΔQ/Δt is the permeation rate of the molecule (the slope of the curve described by the cumulative concentration at the analyzed timepoints), C_0_ is the initial concentration in the donor compartment, and A is the surface area.

To evaluate the retaining ability of the barrier after longer intervals, the same dextran solution was left in the apical compartment for 24 h. Then, 100 µL of solution was collected from both apical and basolateral compartments, and the concentrations were calculated as described above. Data were processed to calculate the percentage of FITC–dextran present in the two compartments.

#### 2.6.2. Quantitative Measurement of Labeled Glucose and Proline in the Culture Medium

To determine the permeability of small molecules of the epithelial barriers formed on the different platforms, we calculated the apparent permeability (P_app_) of D-glucose-6,6-d2 (D2-Glucose, Merck, Darmstadt, Germany, cat. no. 282650) and L-proline-2,5,5-d3 (D3-Proline, Merck, Darmstadt, Germany, cat. no. 791261). The molecules were diluted in the culture medium to a final concentration of 918 and 75 µg/mL, respectively, and added to the apical chamber. After 30, 60, and 120 min, 100 µL of solution was collected from the basolateral compartment and diluted 1:10 with 90/10 (*v*/*v*) acetonitrile/200 mM aqueous ammonium formate at pH 2.8. Samples were centrifuged 10,000× *g* for 10 min, and the obtained supernatant was filtered through a 10 kDa MWCO Omega polyethersulfone UF membrane in a Nanosep Advance device (Pall, Port Washington, NY, USA). Serial dilutions of both molecules were prepared for external standard calibration curves. Controls were performed using inserts without cells.

D2-Glucose and D3-proline were quantified in the samples using ultra-performance liquid chromatography (UPLC) system coupled to a high-resolution Orbitrap mass spectrometer Q Exactive (Thermo Fisher Scientific, San Jose, CA, USA). Detailed instrumentation operative conditions are reported in the Supplementary Materials.

### 2.7. Ultrastructural Analyses with Transmission Electron Microscope (TEM)

Alvetex™ scaffold inserts were fixed in 4% glutaraldehyde in sodium cacodylate buffer 0.2 M for 2 h, followed by a 2 h wash in 0.1 M cacodylate buffer, and then post-fixed with 2% osmic acid in 0.1 M cacodylate buffer for other 2 h. Subsequently, they were dehydrated with a graded scale of ethanol (70%, 90%, 95%, and absolute ethanol). Absolute ethanol was replaced with propylene oxide, and samples were immersed in freshly prepared epoxy-resin solution according to manufacturer’s instructions (Durcupan™ ACM, Merck, Darmstadt, Germany, cat. no. 44611, 44612, 44,613 and 45345), followed by polymerization at 60 °C for 12 h. Semithin sections were cut using an RMC Boeckeler ultramicrotome and stained with toluidine blue. Ultrathin sections were examined using the transmission electron microscope Talos L120C (Thermo Fisher Scientific, San Jose, CA, USA) operating at 120 kV. Images were acquired using a Ceta Camera 4 K × 4 K (Thermo Fisher Scientific, San Jose, CA, USA).

### 2.8. Histochemical Detection of Brush Border Enzymes

To evaluate intestinal cell function, alanine aminopeptidase (ALP), leucine aminopeptidase (LAP), and alkaline phosphatase (AP), three well-defined enterocyte brush border enzymes, were analyzed after TEER reached its plateau in each culture platform. AP, ALP, and LAP detection protocols were first validated on RT intestine sections. Small pieces of RT intestine were promptly snap-frozen in liquid nitrogen and embedded in optimal cutting temperature (OCT) compound. Subsequently, 8 µm thick sections were cut with a Leica CM1500 cryostat (Leica microsystem s.r.l., Wetzlar, Germany). Sections were then post-fixed in precooled acetone for 20 min and air-dried for 10 min. Subsequently, slides were exposed to a solution of 0.1 mL of N,N-dimethlyformamide, 10 mL of 0.1 M cacodylate buffer (pH 7.4), 10 mg of Fast Blue salt, and 5 mg of L-alanine 4-methoxy b-naphtylamide or L-leucine-b-naphtylamide for the detection of ALP or LAP, respectively, for 60 min at room temperature and for 2 h at 4 °C. Subsequently, samples were washed and incubated with 2% copper sulfate for 3 min, post-fixed in 4% glutaraldehyde in 0.1 M cacodylate buffer, and then washed and mounted using a polyvinyl alcohol mounting media. For the in vivo detection of AP, small proximal intestinal fragments were fixed in 10% neutral buffered formalin (NBF) shaking for 24 h at room temperature. They were then dehydrated in graded alcohols, cleared with xylene and embedded in paraffin. Next, 5 µm thick sections were rehydrated and brought to distilled water, and then samples were immersed in fresh Tris—HCl (pH 9.5) solution for 5 min to generate the adequate alkaline environment. Subsequently, slides were exposed to 5-bromo-4-chloro-3-indolyl phosphate/nitroblue tetrazolium (BCIP/NBT (Vector Laboratories, Newark, CA, USA, cat. no. SK-4500) substrate, rinsed in tap water, counterstained using Mayer’s hematoxylin, dehydrated, and permanently mounted.

Images were acquired using a Leica DMR microscope (Leica Microsystems, Wetzlar, Germany) equipped of a Nikon DS-Ri2 camera (Nikon, Amstelveen, The Netherlands) and the NIS-Elements D software, version 5.20.

Similar procedures, with only few adjustments, were applied to detect ALP, LAP, and AP on in vitro samples.

Images were acquired using a V16 stereomicroscope (Carl Zeiss AG, Oberkochen, Germany) equipped with an Axiocam 506 color camera (Carl Zeiss AG, Oberkochen, Germany) and the ZEN 2 version 2.0 package software.

### 2.9. Enzymatic Activity: Alkaline Phosphatase (ALP) and Alanine Aminopeptidase (ALP) In Situ Assay

In situ detection of enzyme activity in live cells in vitro was performed according to the protocol described by Ferruzza [22]. Briefly, culture inserts were washed three times in PBS supplemented with 1 mM CaCl_2_ and 1 mM MgCl_2_ pH 7.0 (PBS^+^). For the detection of alanine aminopeptidase, samples were subsequently incubated with 5 mM L-alanine 4-nitroanilide hydrochloride solution diluted in 10 mM Tris-HCl pH 8.0, 150 mM NaCl in PBS. After 50 min, 100 μL of solution was collected from the apical compartment and transferred to a 96-well plate. Enzyme activity was quantified measuring the optical density at 405 nm (OD_405_) with a microplate reader (BioRad, Hercules, CA, USA, Model 680). The standard curve was prepared from 1 mM p-nitro anilide (*p*-NA) stock solution.

For alkaline phosphatase (AP) detection, inserts were washed three times with PBS^+^, and cells were later exposed to p-nitrophenyl phosphate (*p*-NPP, Single Reagent Yellow Alkaline Phosphatase Substrate, Merck, cat. no. ES009), which, in the presence of alkaline phosphatase enzyme, is hydrolyzed to p-nitrophenol (*p*-NP), a yellow soluble product. After 8 min, 100 μL of solution was collected from the apical compartment and transferred to a 96-well plate on ice, containing 50 μL/well of 0.5 M NaOH to stop the reaction. ALP activity was quantified measuring the OD_405_, and the standard curve was prepared with 50 μL of serial *p*NP concentrations (0–600 μM) added to 150 μL of *p*NPP solution and 50 μL of 0.5 M NaOH.

### 2.10. Statistical Analysis

Results were expressed as the means ± standard deviations. Statistical analysis was performed with SPSS statistics software (version 28.0.0.1, IBM, Armonk, NY, USA) using one-way ANOVA followed by post hoc Tukey’s test, with the only exception being for the analysis of deuterated compounds, which required the Kruskal–Wallis test due to the non-Gaussian distribution of the data. *p*-Values < 0.05 were considered significant.

## 3. Results

### 3.1. Morphological Characterization

#### 3.1.1. Light Microscopy and Immunohistochemistry

A number of morphological analyses of all four organotypic cell-based intestinal platforms were performed. Unfortunately, it was not possible to perform all of them on the MM platform, because its physical properties prevented the adhesion of paraffin-embedded sections on the slides, resulting in the repeated loss of the samples.

**Figure 2:**
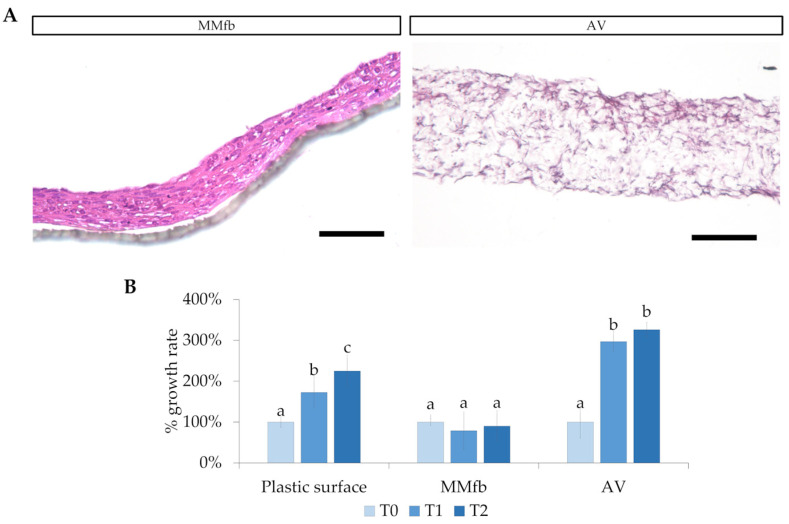
(**A**) Fibroblast colonization and infiltration in the Matrigel matrix (MM) and inside the Alvetex™ (AV) scaffold (HE staining; scale bar 250 µm). (**B**) Relative RTpiMI epithelial cell growth rate, expressed as a count of nuclei. Given the difference among the systems, different timing was selected: for plastic surface and MMfb, T0, T1, and T2 correspond to immediately, 48 h, and 72 h after seeding, respectively; for AV scaffolding, T0, T1, and T2 represent 14, 21, and 28 days, respectively. In all cases, images were acquired at 40× magnification. Different letters within the graph indicate statistically significant differences (*p* < 0.05).

Histological analyses revealed that RTskin01 fibroblasts were able to populate both Matrigel^®^ and Alvetex™; however, HE staining showed that, while fibroblasts grown within the Matrigel^®^ matrix remained very close to each other, they were loosely spread within the Alvetex™ scaffold. Moreover, on both platforms, fibroblasts slowed their proliferation. In particular, when cultured in the MMfb platform, they significantly decreased their doubling time, whereas, in AV, their number increased from day 14 to 21 of culture, and then remained constant during the following week (Figure 2).

**Figure 3:**
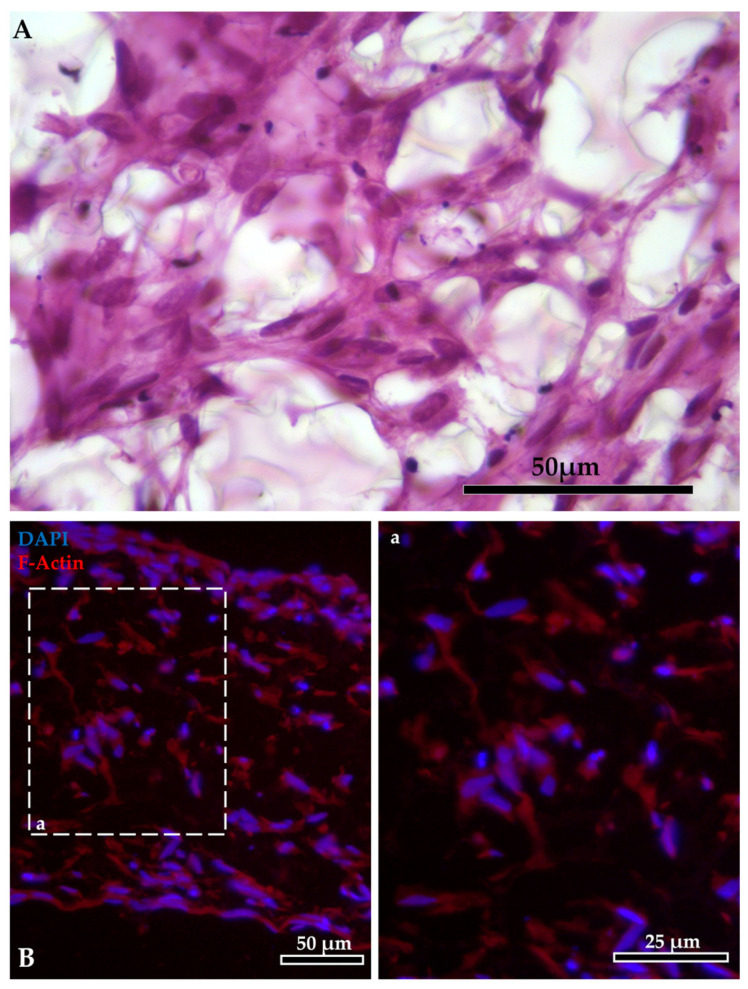
Intricate mesh generated by the fibroblasts inside the Alvetex^TM^ scaffold: (**A**) staining with HE. Fibroblast organization inside the Alvetex^TM^ scaffold: (**B**) F-actin stained in red with phalloidin-iFluor 594 reagent; nuclei counterstained in blue with DAPI (panel a shows F-actin staining at higher magnification).

HE-stained sections and F-actin staining showed that fibroblasts growing within the AV scaffold maintained their native shape and their 3D morphology, generating a network that resembled the stromal environment in vivo (Figure 3).

**Figure 4:**
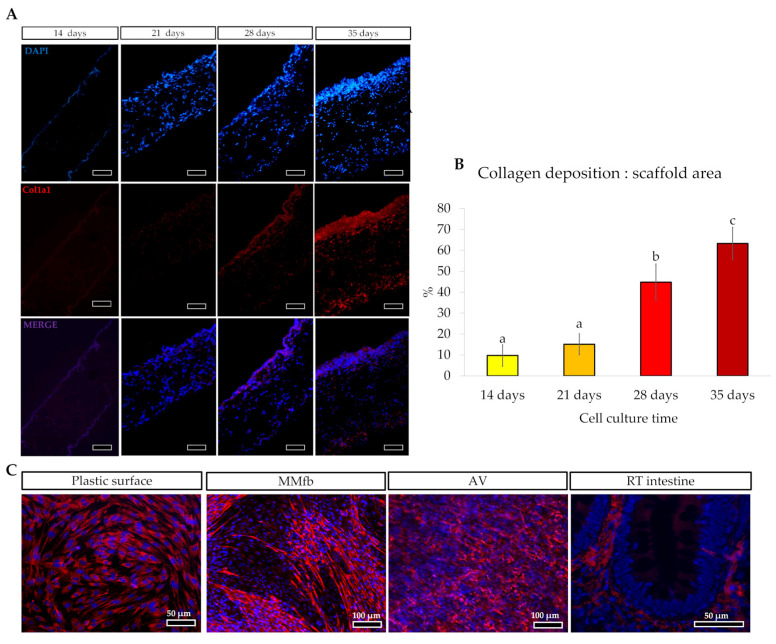
(**A**) Immunodetection of Collagen type 1α1 chain (Col1alpha1; stained in red) in Alvetex™ scaffold showing that fibroblasts started to synthesize and release collagen after 21 days of culture, generating a thick layer of collagen after 28 days (scale bar 50 µm). The thick collagen layer was further expanded after an additional week of culture. (**B**) Bar charts showing that the collagen deposition relative to the total Alvetex™ scaffold area increased in parallel with the cell culture time. Different letters within the graph indicate statistically significant differences (*p* < 0.05). (**C**) Representative images of immunodetection of Col1alpha1 (in red) in fibroblast cultured on flat plastic surface, embedded in Matrigel matrix^®^, cultured inside the Alvetex scaffold and in RT intestinal wall. Images showed that, when RTski01 was cultured on plastic or in Matrigel matrix^®^, Col1alpha1 expression was restricted to the cell cytoplasm; however, when cultured in Alvetex™, RTskin01 fibroblasts produced and released collagen within the extracellular space, generating an artificial stroma resembling the in vivo connective tissue.

Moreover, immunodetection of collagen type 1 α1 chain demonstrated that RTskin01 fibroblasts started to synthetize and release collagen after 21 days of culture. Its deposition increased concurrently with the cell culture time. Furthermore, after 28 days of growth, fibroblasts produced a collagen layer on the Alvetex™ scaffold’s top, which became thicker after a further 7 days (Figure 4). Immunostaining of collagen type 1 α1 chain showed that the fluorescence signal was not restricted to the cell cytoplasm, as occurred when fibroblasts were cultured on standard plastic surfaces but extended in the extracellular space, generating an intricate supportive mesh closely resembling a typical collagen extracellular matrix.

**Figure 5:**
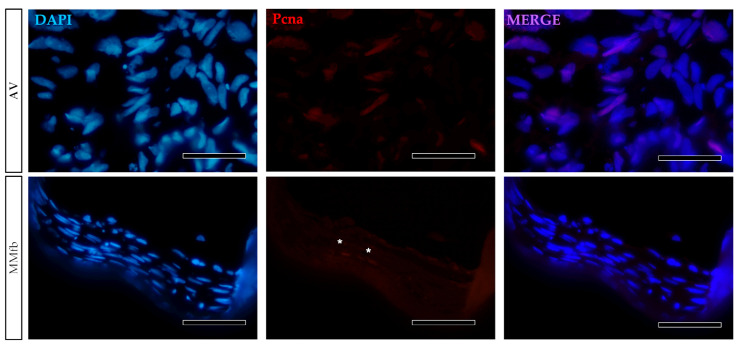
Representative images of Pcna immunodetection (red) showing that only a few RTskin01 fibroblasts grown into Alvetex™ (AV) or Matrigel^®^ with fibroblasts (MMfb) were actively dividing. Nuclei were counterstained with DAPI (scale bar 50 µm) (white asterisks in the MMfb panel, indicate Pcna^+^ cells).

The presence of Pcna in only a few fibroblasts grown on MM and AV scaffolds confirmed that most of them arrested their growth when cultured in combination with a 3D biological or synthetic matrix (Figure 5).

The classic epithelial layer grown directly on a PET culture insert (TC) was compared with more complex platforms made of two cell types: the fibroblasts seeded within the Matrigel^®^ and Alvetex™ scaffolds combined with the intestinal epithelial cells seeded on top. Due to the high porosity of the AV scaffold, epithelial cells were seeded only after the generation of a thick collagen layer underneath the apical surface of the scaffold to avoid their infiltration inside the scaffold itself.

**Figure 6:**
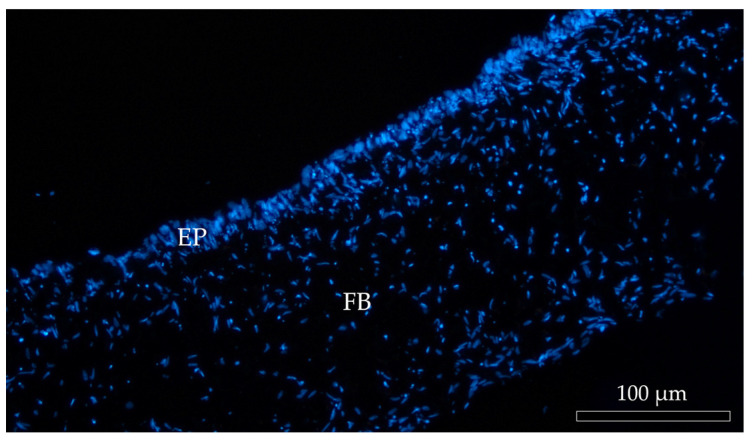
Representative image of DAPI-stained (blue) cell nuclei of Alvetex™ scaffold populated with fibroblasts (FB) creating an anchorage site for the epithelial cells (EP).

**Figure 7:**
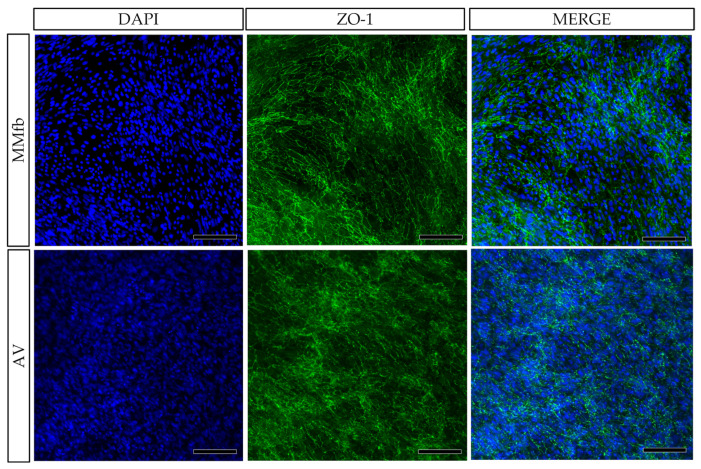
Immunodetection of ZO1 (green) showing the presence of a homogenous layer of epithelial cells on the top of the organotypic platforms: TC inserts with Alvetex™ (AV) and with Matrigel^®^ enriched with fibroblasts (MMFb). Nuclei were counterstained with DAPI (blue) (scale bar 200 µm).

In all culture conditions, RTpiMI epithelial cells completely covered the apical surface of the inserts. Moreover, HE and DAPI staining showed the presence of a uniform layer of tightly aligned epithelial cells covering entirely the apical surface (Figure 6). The presence of a homogenous layer of adherent epithelial cells was also confirmed by the immunolocalization of the junctional protein zonula occludens 1 (ZO1) (Figure 7).

**Figure 8:**
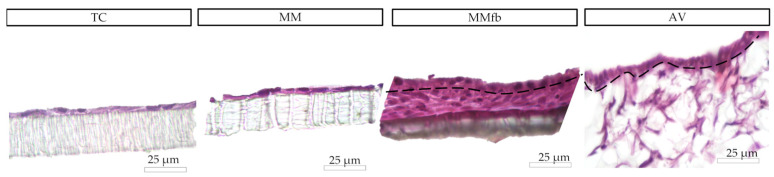
Organization of epithelial cells and fibroblasts on different platforms (HE staining). Epithelial cells displayed a flat and elongated shape when cultured on the TC insert alone or with a thin Matrigel matrix^®^ coating (MM). When epithelial cells were cocultured with fibroblasts embedded in Matrigel^®^ (MMfb), they acquired a cubic phenotype that became more cylindrical in the AV.

**Figure 9:**
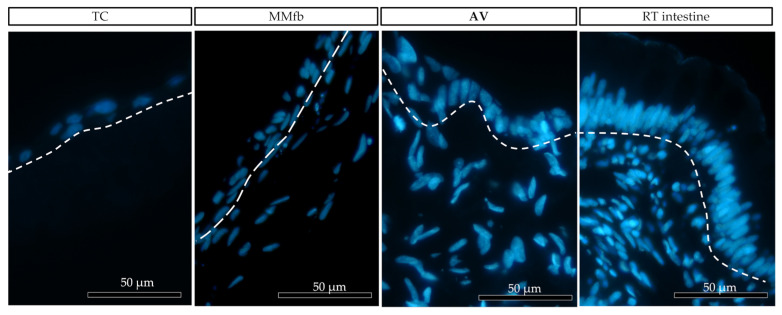
Representative images of the organization of epithelial (above the dotted line) and fibroblast cell (below the dotted line) growth on the different platforms: ThinCert^®^ (TC) inserts, Matrigel Matrix^®^ enriched with fibroblasts (MMfb), Alvetex™ scaffold (AV), and the RT intestine. Nuclei were stained with DAPI (in blue). The three platforms showed an increasing similarity (from left to right) with their in vivo counterpart.

**Figure 10:**
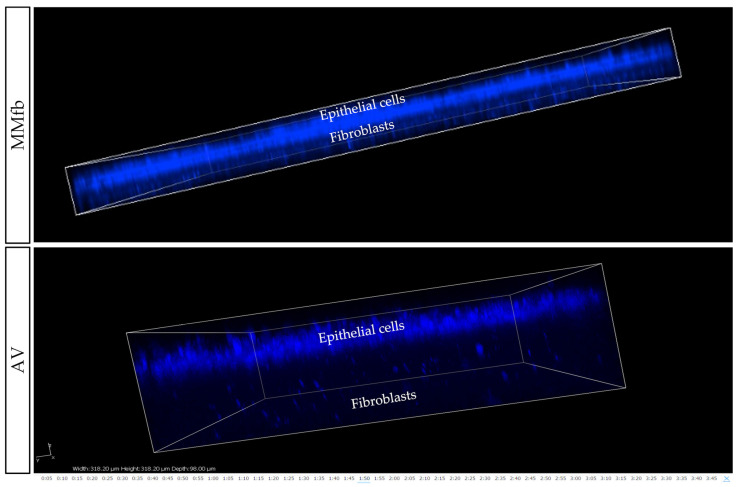
Representative confocal microscopy images of the MMfb and AV scaffolds. Epithelial cells on both platforms entirely covered the apical surface, while the fibroblasts beneath remained distinct and showed the typical organization found in the connective tissue. Nuclei were stained with DAPI (blue).

After 7 days, on all platforms, HE- and DAPI-stained paraffin sections (Figure 8 and Figure 9) revealed that RTpiMI cells were organized in a cell monolayer. Epithelial cells grown on TC and MM showed a flat and elongated shape, whereas when cultured with MMfb and AV, epithelial cells acquired a cubic phenotype, that became more cylindrical in AV from day 21 of culture. In both cases, epithelial cells and fibroblasts formed two contiguous layers but did not mix. The distribution of the epithelium, above, and the stroma, beneath, was confirmed by confocal microscopy images (Figure 10).

**Figure 11:**
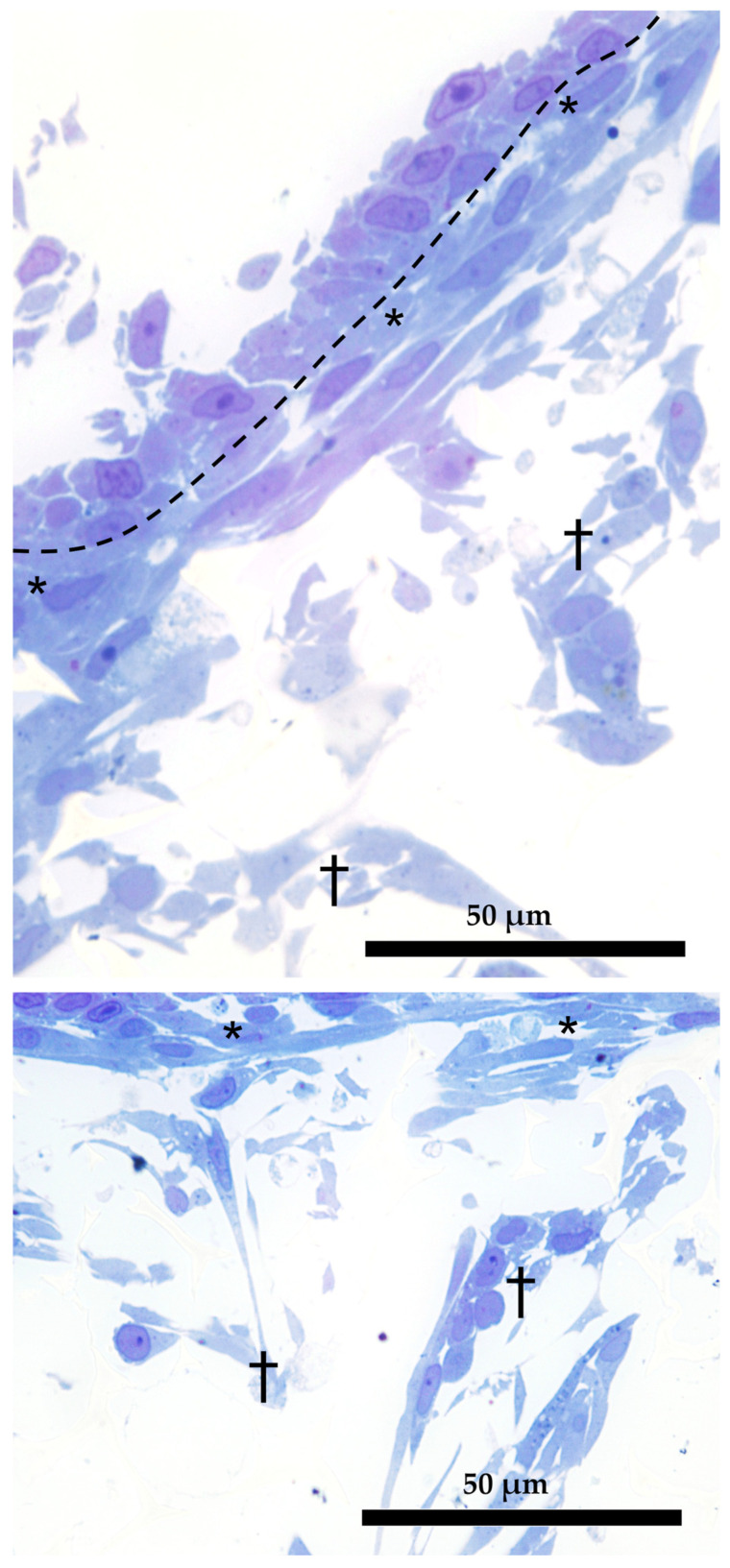
Dual organization of RT fibroblasts within the AV scaffolding (semithin section stained with toluidine blue). Fibroblasts in the close proximity of the epithelial cells assumed a thin and elongated morphology (asterisks); those located more in-depth acquired a loose distribution and a varied phenotype (crosses). Dotted line underlies the boundary between epithelial and fibroblasts cells.

Semithin sections showed that the morphology of RTskin fibroblasts cocultured with epithelial cells in the AV was different depending on their localization within the scaffold. Specifically, those located in close proximity to the epithelial cells assumed a thin and elongated shape resembling the cell population that in vivo is regularly aligned just below the basal membrane [10]; conversely, those distributed more in-depth within the scaffold acquired a spaced out distribution and varied phenotype (Figure 11).

**Figure 12:**
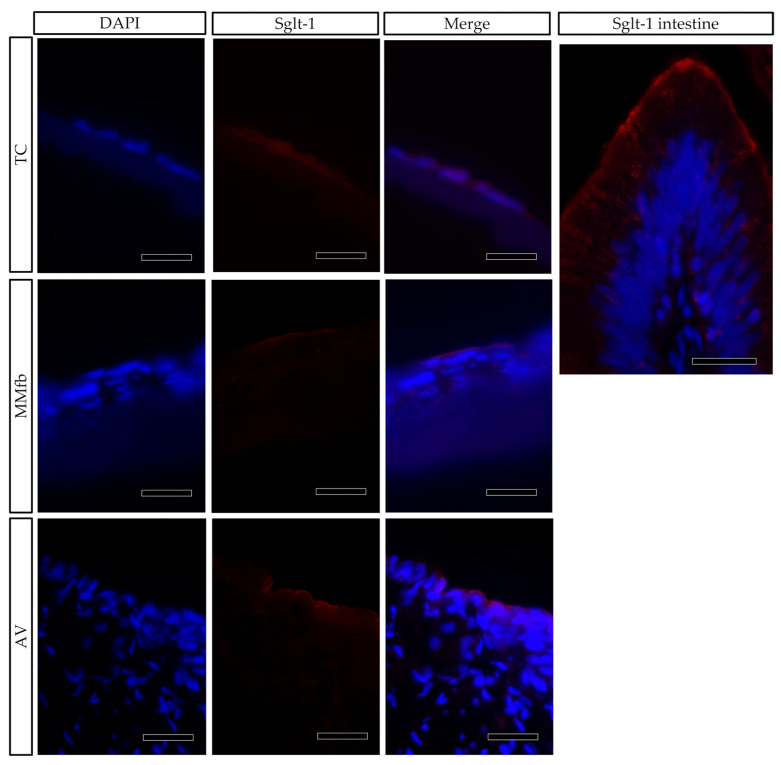
Immunofluorescence staining of Sglt1 (red) showing its distribution in epithelial cells grown on ThinCert^®^ inserts (TC) or fibroblasts embedded in Matrigel^®^ (MMfb), Alvetex™ scaffold with fibroblasts (AV) and in the RT intestine (Sglt-1 intestine) (scale bar 25 µm). Nuclei were counterstained with DAPI.

Sodium–glucose/galactose transporter 1 (Sglt-1) was used as a marker of cellular polarization. In the RT intestine, the Sglt-1 signal was localized along the enterocyte brush border [9], but it was spread in the cell cytoplasm when RTpiMI epithelial cells were cultured on TC inserts. However, its localization was restricted to the apical surface when epithelial cells were grown in MMfb and AV (Figure 12).

#### 3.1.2. Ultrastructural Analyses

Since the AV platform showed the best results in terms of cell morphology and cell distribution, we also examined the samples with the transmission electron microscope (TEM) for a more detailed characterization of the intracellular structures.

**Figure 13:**
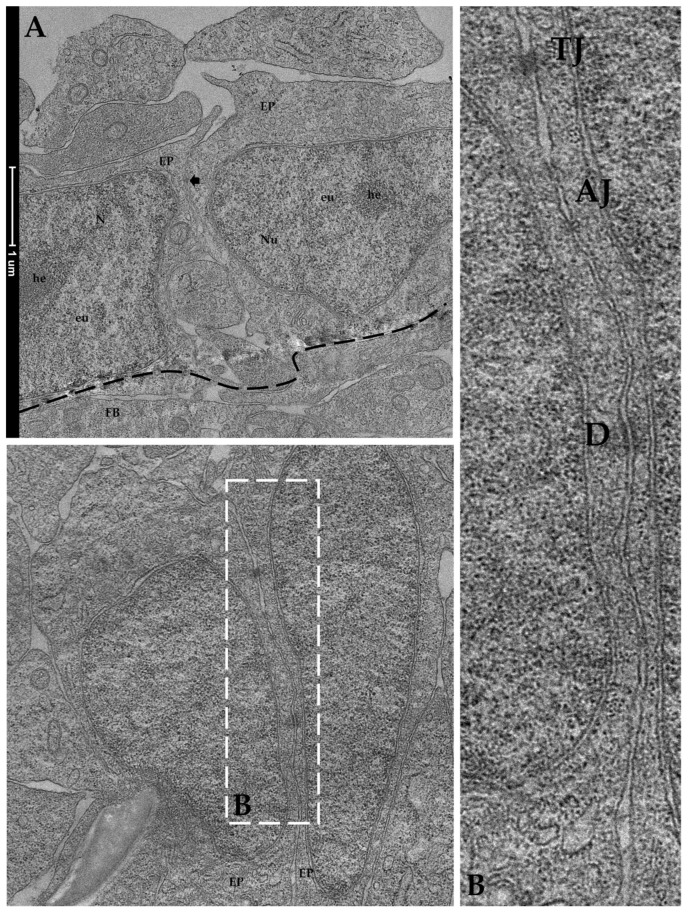
Representative TEM images of the organization of RTpiMI epithelial cells cocultured with RTskin01 fibroblasts in AV scaffold showing tightly aligned epithelial cells (arrow), with cell nuclei characterized by densely packed heterochromatin cluster and supported by fibroblasts: (**A**) EP epithelial cells; Nu: nucleus, FB: fibroblast cell; he: heterochromatin; eu: euchromatin. At higher magnification, tight junctions, adherens junctions, and desmosomes are visible between neighboring cells: (**B**) tight junctions: TJ; adherens junctions: AJ; desmosomes: D.

**Figure 14:**
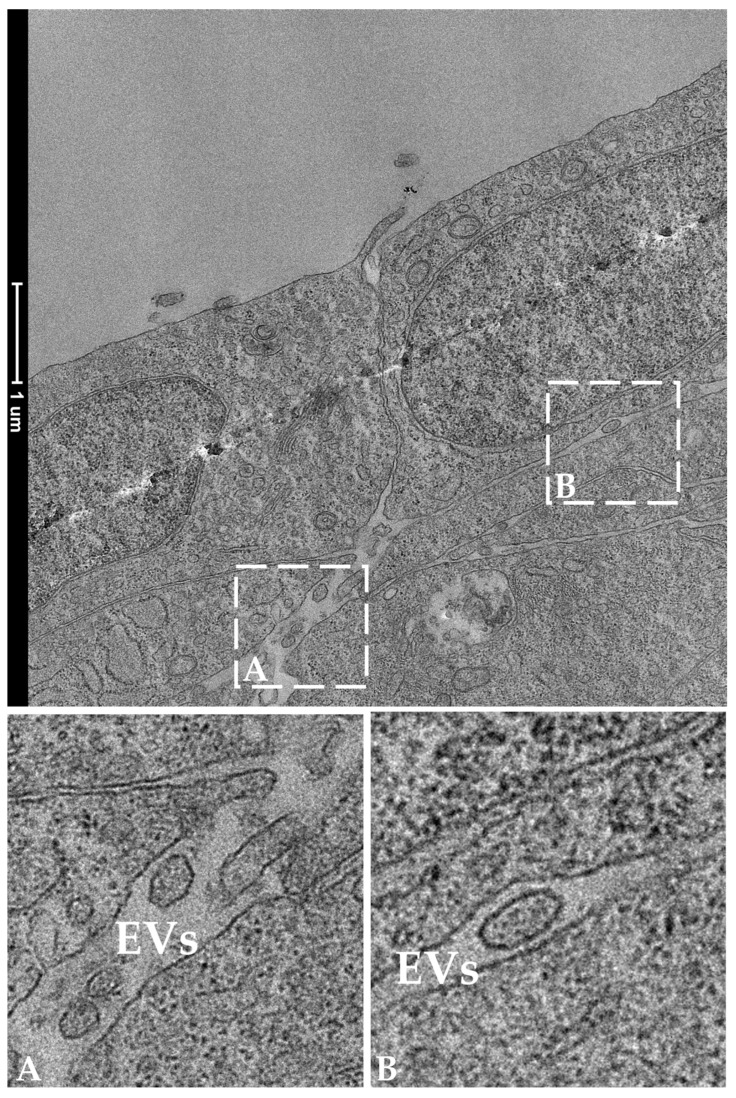
Representative TEM images showing the presence of extracellular vesicles (EVs) in the extracellular space in the close proximity of two epithelial cells. Panels A and B show EVs at higher magnification.

TEM analysis performed on RTpiMI cells cultured for 21 days on AV confirmed the absence of microvilli but showed the presence of a complete set of junctional complexes between adjacent epithelial cells (Figure 13). Those located in the more apical region and defined by a dark dense band were identified as tight junctions (TJs), those distributed in the intermediate regions were classified as adherens junctions (AJs), and those located in the basal portions of neighboring cells that appeared as dark interrupted bands were recognized as desmosomes (D) [23]. Moreover, epithelial cell nuclei were characterized by densely packed heterochromatin clusters, a typical indicator of mature enterocytes [10]. Furthermore, TEM analyses highlighted the presence of peculiar spherical structures encircled by a well-defined biological membrane ascribable to extracellular vesicles. They were distributed scattered among fibroblasts within the extracellular space (Figure 14).

### 3.2. Functional Characterization of the Epithelial Barrier

#### 3.2.1. TEER Measurements

TEER was monitored during the culture period to detect the formation of a functional epithelial barrier in vitro that was achieved when a plateau value was attained. The TEER plateau value was different for each platform with ~40 Ω·cm^2^ for TC on day 7), followed by MM (~45 Ω·cm^2^), MMfb (~70 Ω·cm^2^), and AV (~85 Ω·cm^2^). The latter two leveled up on day 21. As expected, MMfb and AV scaffolds, cultured with RT skin fibroblasts but without the epithelial cells, did not develop any electrical resistance. Given the variability among the different platforms, to properly compare them, we performed all other functional analyses only after the attainment of the TEER plateau.

#### 3.2.2. Permeability Assays

**Figure 15:**
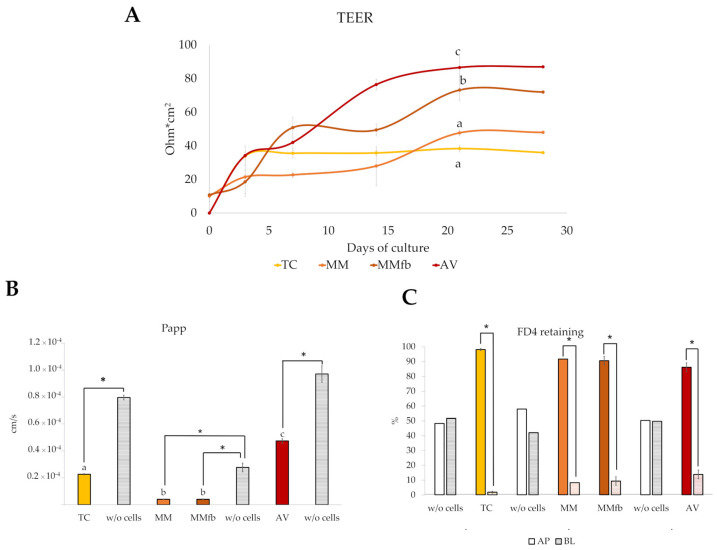
Functional assessment of the epithelial barrier properties culturing RTpiMI cells on different platforms (TC: transwell insert as control; MM: Matrigel^®^ with epithelial cells only; MMfb: fibroblasts embedded in Matrigel^®^ and epithelial cells; AV: Alvetex™ with fibroblasts and epithelial cells). (**A**) TEER measured along 28 days of culture. (**B**) Apparent permeability of the paracellular flux of 4 kDa FITC–dextran (FD4) estimated after the TEER values reached the plateau. (**C**) More than 90% of FD4 was retained in the apical compartment of all platforms after 24 h incubation, indicating the attainment of a functional epithelial barrier able to prevent the paracellular flux of relatively large molecules. This did not occur in the absence of epithelial cells (*w*/*o* cells), confirming the specificity of the effect. AP, apical compartment; BL, basolateral compartment Values are expressed as means ± SD. Different letters or asterisks within the same graph indicate statistically significant differences (*p* < 0.05).

The generation of a functional barrier in vitro was also evaluated by the ability of epithelial cells to retain large molecules in the apical compartment, preventing the paracellular flux through the epithelial cell layer. After each platform reached its TEER plateau value, the platforms were exposed to 4 kDa FITC–dextran (FD4), and the apparent permeability through the barrier system was measured during a 2 h period. In all culture conditions, the FD4 flow rate through the barrier system cultured with RTpiMI epithelial cells was significantly lower compared to the inserts without cells. TC, MM, and MMfb showed a significantly lower flow rate compared to AV. We also measured the percentage of FD4 in the apical and in the basolateral compartment after 24 h of incubation to verify whether the epithelial cells could retain large molecules for longer intervals on the different platforms. After 24 h, on all platforms, the percentage of FD4 retained in the apical compartment was around 90%, while, in the absence of cells, FD4 was close to the equilibrium (Figure 15).

#### 3.2.3. Enzymatic Activity

**Figure 16:**
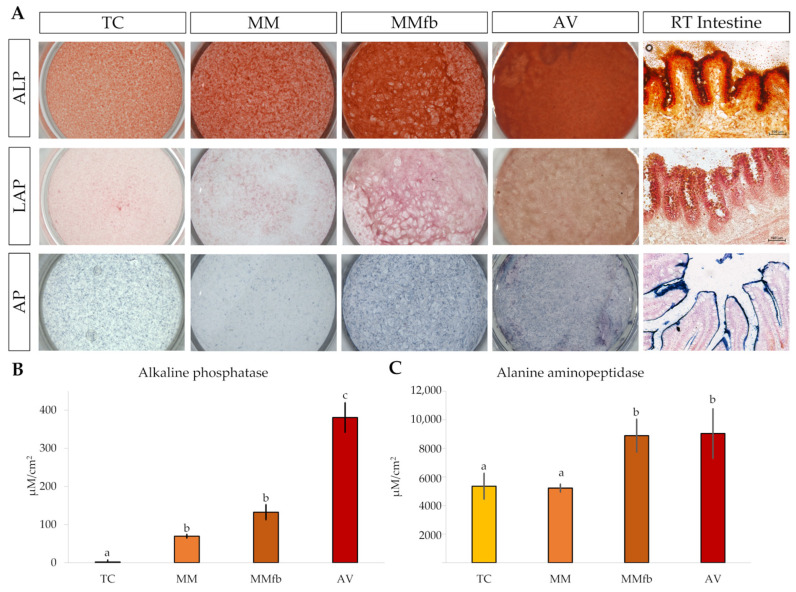
(**A**) Representative images showing the enzymatic activity of L-alanine aminopeptidase, L-leucine aminopeptidase, and alkaline phosphatase in RTpiMI cells cultured on ThinCert^®^ (TC) inserts, on TC coated with Matrigel^®^ (MM), on MM embedded with fibroblasts (MMfb), on Alvetex™ scaffold (AV), and in the RT intestine. Quantification of the enzymatic activity of alkaline phosphatase (**B**) and alanine aminopeptidase (**C**) in RTpiMI epithelial cells cultured in combination with increasingly complex in vitro platforms. Values are expressed as means ± SD. Differences were considered statistically significant if *p* < 0.05 (different superscripts within the same graph indicate statistically significant differences).

The histochemical detection of alanine aminopeptidase (ALP), leucine aminopeptidase (LAP), and alkaline phosphatase (AP) showed an increasing signal gradient from the simpler to the more complex platforms. Indeed, signals were low in TW insert, intermediate when RTpiMI cells were cultured in combination with MM, higher in MMfb, and highest in the AV scaffold (Figure 16A).

However, since the histochemical signal intensity is not linear and, thus, does not provide an accurate quantification, the enzymatic activity was measured through in situ detection assays on live cells that, on the contrary, can be accurately quantified. Interestingly, AP measurements confirmed the gradual significative increase from TC to AV, whereas ALP activity was significantly higher when RTpiMI cells were cultured in combination with fibroblasts in the MMfb and AV scaffolds, compared to epithelial cells grown alone on TC and MM insert (Figure 16B,C).

#### 3.2.4. Transport of D2-Glucose and D3-Proline across the Epithelial Barrier

**Figure 17:**
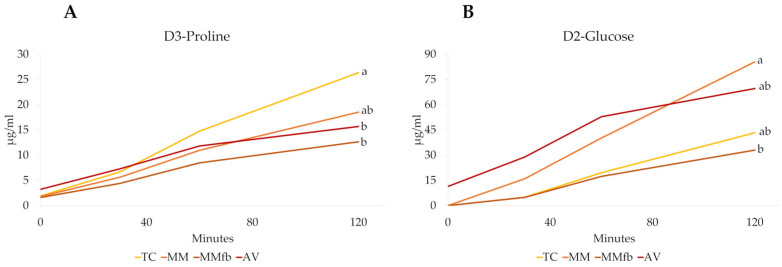
D3-Proline (Panel (**A**)) and D2-glucose (Panel (**B**)) transport over time (0 to 120 min) through the epithelial barrier seeded on the different platforms: the ThinCert^®^ culture inserts (TC); TC inserts coated with Matrigel^®^ (MM); TC inserts coated with Matrigel^®^ enriched with fibroblasts (MMFb); Alvetex™ scaffold enriched with fibroblasts (AV). Different letters within the same graph indicate statistically significant differences (*p* < 0.05).

The transepithelial transport of D3-proline and D2-glucose labeled molecules was measured to compare the capacity of platforms to promote the active transport of nutrients across the epithelial barrier. After each platform reached its TEER plateau, D3-proline and D2-glucose were added to the apical chambers, and the transepithelial flux through the barrier was calculated during a 120 min exposure (Figure 17). The rate of transport from the apical to the basolateral chamber varied among the different platforms and between the two molecules.

## 4. Discussion

In the present work, we developed and compared four cell-based organotypic rainbow trout intestinal in vitro platforms. The simplest was formed by epithelial cells directly layered upon a PET membrane with 0.4 µm pores within ThinCert^®^ inserts (TC). To this basic structure, we added a thin layer of Matrigel^®^, a solubilized mouse basement membrane isolated from Engelbreth–Holm–Swarm sarcoma cells and made of laminin, collagen type IV, proteoglycans, and growth factors, and we used a PET membrane with 3 µm pores (MM). Alternatively, the epithelial cells were layered upon the same membrane but coated with fish fibroblasts embedded in Matrigel^®^ (MMfb). Lastly, epithelial cells were layered on Alvetex™, a highly porous polystyrene insert that we populated with fish fibroblasts synthesizing fish extracellular matrix (AV). Our aim was to identify the platform that would best stimulate the differentiation of intestinal cell lines toward the mature enterocyte phenotype. We performed several morphological staining experiments; however, we were unable to carry out all of them on the MM platform, which frequently resulted in sample loss. This difficulty is likely due to the disadvantageous ratio between the forces of adhesion over the face of the tissue and the perimeter forces pulling off the tissue from the surface area [24], caused by the thin layer of cells and the thick layer of Matrigel, respectively. In the future, it will be worth testing some alternative adhesives such as protected isocyanate [25] or adding small scalpel nicks perpendicular to the tissue so that some extra points of adhesion are created [24]. RT fibroblasts successfully populated both scaffolds, but they were densely distributed within MMfb, whereas they were evenly spaced in AV. HE and F-actin staining confirmed that fibroblasts in AV preserved their native shape and typical 3D disposition, a very desirable feature when reproducing an in vivo-like platform. This observation demonstrates that 3D culture systems promote proper cell-to-cell interactions, as well as an in vivo-like spatial organization [26]. In the early culture stages, fibroblasts were preferentially located in the AV peripheral regions. However, after 21 days of culture, cells populated the whole scaffold thickness, indicating that AV supported cell adhesion and growth. This is consistent with previous studies performed using the same scaffold in combination with primary dermal fibroblast or with a fibroblast colonic cell line (CCD-18co) [27]. Immunostaining of proliferating cell nuclear antigen (Pcna) reveled that only a few fibroblasts were actively dividing on both MMfb and AV platforms, as opposed to the active proliferation commonly observed in standard tissue culture flasks [28]. This further confirmed that an enriched environment discourages cell proliferation while promoting differentiation and extracellular matrix deposition. Indeed, in the adult body, fibroblasts are mostly found in a quiescent state [29] unless local stimuli promote their migration and activate protein synthesis.

Immunodetection of collagen 1 alpha 1 (Col1a1) demonstrated that RT fibroblasts cultured in AV synthesized and deposited collagen, creating their own matrix. This was previously observed also for human fibroblasts grown both in AV [14] and in a collagen layer [30] where they secreted laminin and fibronectin, the two major non-collagenous matrix glycoproteins. Unfortunately, due to the lack of antibodies specific for RT, we were not able to verify whether these molecules were also produced in our fish model. Interestingly, Col1a1 was not restricted to the cell cytoplasm, as occurred when RT fibroblasts were cultured on standard plastic surfaces, but it was secreted in the extracellular space, generating an intricate supportive mesh closely resembling the typical extracellular matrix found in vivo. Collagen deposition increased during the culture period, emphasizing the active role of fibroblasts in remodeling the extracellular matrix over time. Additionally, after 21 days, RT fibroblasts created a thick layer of collagen in correspondence to the scaffold’s apical surface. As previously reported [14], the formation of this layer is considered a reliable marker indicating the right time for epithelial cells to be seeded, and our experience confirms this observation. RTpiMI epithelial cells successfully covered the inserts apical surface, forming a regularly aligned cell layer in all culture conditions, including AV, despite it presenting pores large enough for the epithelial cells to go though. This indicates that the collagen layer synthesized by RT fibroblasts properly sustained the overlaying epithelium. Inert scaffolds surfaces are commonly precoated with different biological matrices [31,32] to promote cell adhesion and to generate a stiffness level that replicates the features of the native organ. In this experiment, the natural collagen produced by RT fibroblast seemed to provide the optimal biomechanical cues to encourage epithelial cell adhesion.

After 7 days of culture, on all platforms, RTpiMi cells were organized in a monolayer, a distinctive feature of the intestinal epithelium. Interestingly, while epithelial cells on the TC and MM inserts appeared flat and elongated, on the more complex MMfb and AV platforms, epithelial cells acquired a more cubic phenotype that in AV became cylindrical after 21 days of culture. The same observation was recently reported for human intestinal cells grown with AV, and it was suggested that the presence of ECM components stimulates epithelial cells to acquire an in vivo-like columnar phenotype [14,29]. Our observations support this hypothesis, and further suggest that the better effect provided by AV may be attributed to the homospecific ECM synthesized by rainbow trout fibroblasts, as opposed to Matrigel^®^, which is of mouse origin.

The shape of RT fibroblasts cultured in the AV platform differed depending on their spatial position within the scaffold. Fibroblasts close to the epithelial cells assumed a thin and elongated shape strongly resembling the subepithelial cell population that, in vivo, is evenly distributed just below the enterocyte basement membrane [10]. In vivo, this specific cell population establishes a direct connection with the overhead epithelium and allows the physiological preservation of the intestinal architecture. On the basis of this observation, it is plausible to assume that something similar happens also on our intestinal platforms, as further discussed below.

Sodium–glucose/galactose transporter-1 (Sglt-1) is the protein responsible for the active transport of glucose though the enterocyte membrane in the small intestine [33]. We previously demonstrated that, in RT, its signal is restricted to the enterocyte brush border [9]. Immunolocalization of Sglt-1 demonstrated that, on both MMfb and AV platforms, epithelial cells acquired an appropriate polarization given that the signal was limited to the apical surface as in vivo. Conversely, when epithelial cells were cultured in TC, the Sglt-1 signal was diffused throughout the cytoplasm. This expression pattern fully matched that observed when RTpiMI cells were cultured onto a plastic surface and suggests a lower degree of differentiation.

Zonula occludens-1 (ZO-1) is a structural protein of tight junctions. In vivo, it interacts with other proteins belonging to the classes of cadherin and claudin contributing to the maintenance of a functional epithelial barrier and to signal transduction [34]. Therefore, ZO-1 immunodetection indicated the formation of a tightly aligned epithelium and a functional barrier. Indeed, the capability of epithelial cells to generate a functional barrier in vitro is commonly investigated by measuring the transepithelial electrical resistance (TEER), as reported in rat, human, and several domestic species such as pig, poultry, and rainbow trout [35]. TEER values increased from baseline levels to plateau on all organotypic platforms, albeit at different timepoints depending on the platform, indicating that a functional barrier was formed in all cases. TEER plateau values with MMfb were higher than MM or TC. This indicated that the presence of a basement lamina, with or without fibroblasts, was required for increasing the TEER. This observation agrees with the results described in a recent work where the authors showed that higher TEER values were recorded only when RT epithelial cells were separated from the fibroblasts by an alumina membrane, whereas the direct contact between the two cell types or the presence of only epithelial cells generated significantly lower TEER values [19]. Interestingly, in our experiments, epithelial cells grown on the AV platform generated the highest TEER values. This suggested that the presence of a basement lamina secreted by fibroblasts of the same species was even more efficient in stimulating cell differentiation. It is difficult to interpret the true physiological meaning of these data. TEER values in fish intestinal epithelia range mostly between 30 and 200 Ω/cm^2^ [36]. However, the electrical resistance in vivo is measured on intestinal explants, consisting of the whole wall and using the Ussing chamber [37]. This implies a procedure different from that used for a cell monolayer, making it difficult to directly correlate in vivo and in vitro values. Nevertheless, our results are consistent with the findings described in studies performed using the human intestinal cell line Caco-2, showing that TEER values in 3D coculture models were closer to those recorded in the human small intestine than to those in simple cell inserts [14].

To further confirm the presence of an effective barrier in vitro, and to evaluate whether the increased TEER values correlate with the ability of epithelial cells to prevent the paracellular flux of large molecules, we analyzed the apparent permeability of 4 kDa FITC–dextran (FD4) along a 2 h exposure, after the TEER reached its plateau on all platforms. As expected, in all culture conditions, when cells were present, the FD4 flux rate was significantly slower than without cells, confirming the ability of RTpiMI cells to form a functional barrier. Our results are consistent with those reported for human intestinal cells grown on Alvetex™ [14] and with those reported for RT intestinal cells cultivated on PET inserts [36]. However, in our work, RT cells cultured on AV showed a flux rate faster than on the other platforms. This could be explained by the fact that the PET inserts used in our experiments had a smaller pore size (0.4 or 3 µm) than Alvetex (36–40 µm) and, in the case of MM and MMfb, were coated with solubilized basement membrane matrix. These different options may alter the transport of molecules across the system after they have passed through the barrier. This hypothesis would be consistent with the conclusion of a recent thorough comparison between TEER and the flux of high-molecular-weight molecules, highlighting that the two are independent cellular parameters which are regulated independently [38]. Therefore, the two techniques should be used simultaneously, especially when characterizing previously unexplored experimental setups.

In vivo, the intestinal epithelium acts as the first layer of defense between the lumen and the vascular system. This complex function is ensured by the presence of intercellular junctions, multiprotein complexes that maintain epithelial cells tightly aligned while promoting the transport of ions, nutrients, and water [39]. Indeed, any alteration of the intercellular junctions is associated with intestinal barrier dysfunction and increased susceptibility to disease [40]. TEM investigation performed on the AV platform demonstrated that epithelial cells developed intercellular junctional complexes between neighboring cells, consistently with previous data reported in another RT intestinal cell line [36] that, however, was derived from the distal intestine. Given the crucial role of these junctions in generating functional barriers in vivo, it was important to find them in vitro. On the basis of their position and peculiar morphological features, it was possible to identify tight junctions (TJs), desmosomes (D), and adherens junctions (AJs) [41]. Tight junctions were located in the most apical region of the cell cytoplasm and were defined by a dark dense band, and desmosomes were localized in the basal region of the epithelial cells and appeared as a dark interrupted band, whereas adherent junctions were distributed in between. Moreover, TEM showed that epithelial cell nuclei were characterized by densely packed heterochromatin clusters that are typical of differentiated cells [10], thus confirming the ability of the AV platform to stimulate cell differentiation.

Within the AV scaffold, we detected spherical structures, each encircled by a well-defined biological membrane. On the basis of their morphological features and position, these spherical structures were classified as extracellular vesicles, a well-characterized intercellular means of communication through which neighboring cells convey proteins, messenger RNA, and microRNA (miRNA) to modulate gene expression and the proliferation or differentiation of the receiving cells [42]. Interestingly, we recently showed that, in the RT intestine, EVs are produced and released in the extracellular space by the telocytes, a specific interstitial cell population that plays a crucial role in modulating intestinal homeostasis and function [10]. The fact that in vitro RT stromal cells also use this means of communication further supports the notion that AV provides conditions that best resemble those found in vivo.

In vivo, intestinal epithelial cells are covered by apical membrane structures known as microvilli that constitute the brush border and act as the anchorage site of several specific digestive enzymes responsible for the final stage of luminal digestion prior to absorption. Among others, enzymes belonging to the oligopeptidase and oligosaccharidase families play a crucial role in the hydrolysis of oligomers after buccal, gastric, and pancreatic digestion [43]. Therefore, brush border enzymes are considered reliable markers of intestinal cellular activity and functionality. Although TEM investigation did not show the presence of microvilli on the apical surface of the epithelial cells, gene expression analysis revealed that RTpiMI cells in all culture conditions actively transcribed leucine (LAP) and alanine (ALP) aminopeptidase genes, as we previously described [44]. The histochemical detection of LAP and ALP activity further demonstrated their translation into active enzymes. In vivo, digestive enzymes are synthesized as proenzymes in ribosomes distributed on the surface of the rough endoplasmic reticulum [43], which are then subjected to post-translational glycosylation. Subsequently, they are transferred to the enterocyte apical membranes, allowing the exposure of the catalytic site into the intestinal lumen [43]. Therefore, it is important to detect in vitro not only ALP protein expression, but also its activity, because this suggests that RTpiMI epithelial cells have the capability to go through this complex synthetic process and to participate in protein digestion. ALP activity gradually increased from the simplest to the most complex cell-based organotypic platform, further emphasizing the key role exerted by the culture environment to promote epithelial differentiation. Interestingly, our results are coherent with data performed using Caco-2 cells demonstrating that the activity of ALP significantly increases when epithelial cells are cocultured with a myofibroblast cell line compared to the controls (cell culture inserts cultured with only epithelial cells) [27].We also detected the activity of alkaline phosphatase (AP), an essential enzyme that plays a key role in the maintenance of intestinal homeostasis, as well as the regulation of lipid absorption and bicarbonate secretion [45]. Furthermore, in RT intestine, as in mammals, AP can be considered a specific marker of cell differentiation since it is selectively localized along the fold length and at the fold apex, while it is absent at the fold base, the typical stem/proliferating zone [8]. As for LAP and ALP, AP histochemical detection followed a clear progressive increase in its signal from the simplest platform to the most complex platform. Moreover, in situ detection of AP on live cells further showed that, while AP activity was almost absent in TC, it progressively increased when a thin coating of MM was added and sharply raised when fibroblasts and epithelial cells were cocultured. This observation suggests that fibroblasts not only acted as modulators of epithelial cellular function, but were also actively involved in promoting epithelial differentiation. Furthermore, the fact that, in AV, the AP activity was more than double compared to MMfb strengthens the hypothesis that the ECM components synthesized and deposited by RT fibroblasts within the Alvetex scaffold are more effective than the mouse components contained in the Matrigel, as well as confirms the observation that AV is the platform that most efficiently stimulates epithelial differentiation.

In vivo, most nutrients end up into the portal system through the highly polarized epithelial cells lining the intestinal mucosa [46]. In the current experiments, we observed that the translocation rate of glucose and proline from the apical to the basolateral compartment was different between molecules and among platforms. Among sugars, glucose represents an essential source of energy for living cells. However, its large size and polar nature prevent its passage through the cellular membrane via simple diffusion. Conversely, glucose uptake is strictly regulated by the glucose transporter protein family. While sodium–glucose linked transporters (SGLTs) are localized on the enterocyte brush border and are responsible for the internalization of glucose from the extracellular environment, facilitated diffusion glucose transporters, such as GLUT2, are generally confined to the enterocyte basolateral membrane, allowing the transport of glucose from the cell to the portal system [47,48]. Similarly, in fish enterocytes, the peptide transporter PEPT1 is responsible for the absorption of approximately 80% of digested proteins, in the form of tri- and dipeptides [49,50], while different transporters handle the transit of amino acids across the basolateral membrane [51]. In particular, the absorption of D-proline in vivo seems to be carried out by a specific proline transporter known as IMINO transporter [52]. Our results showed that the translocation of small molecules was influenced by the platform structure with a different pattern for each molecule. We can hypothesize that the final outcome results from the combined effect of the physical properties of the different membranes and their effect on epithelial cell morphology since, in vivo, nutrient absorption mechanisms are governed by epithelial cell polarization and by the asymmetric spatial organization of cellular organelles and membrane specializations [53].

## 5. Conclusions

In summary, the combined presence of fibroblasts and epithelial cells within 3D scaffolds provided by the Matrigel^®^- and Alvetex™-based organotypic platforms showed better morphological and functional characteristics than the simpler platforms. Rainbow trout fibroblasts grown in the Alvetex™ scaffold better preserved their typical morphology and spatial distribution, as well as synthesized and secreted their own ECM, including a thick layer of collagen that acted as an anchorage site for the epithelial cells. This stimulated a more complete differentiation and polarization toward the typical phenotype of the mature enterocytes. All this indicates that AV is the platform that, overall, better recapitulates the morphology and architecture of the intestinal mucosa. The degree of similarity between the functional properties of the platforms and the intestinal mucosa is more complex to evaluate because it is difficult to find reference parameters that can be directly compared.

## Figures and Tables

**Table 1:** Antibody main features.

	Supplier	Cat. No.	Host	Concentration
Col1a1	Antibodies-online	ABIN237021	Rabbit	1:40
Pcna	Merk	MAB424R	Mouse	1:1200
Sglt-1	Merk	07-1417	Rabbit	1:200
ZO-1	Life Technologies	339188	Mouse	1:100

## Data Availability

The data presented in this study are openly available in the Fish-AI repository hosted at https://dataverse.unimi.it/dataverse/fish-AI (accessed on 10 July 2023).

## Supplementary Materials

The following supporting information can be downloaded at: https://www.mdpi.com/article/10.3390/cells12141843/s1, Figure S1: Extracted ion chromatograms of Glc-D2 (A) and Pro-D3 (B) of selected sample. Reference [54] is cited in Supplementary Materials.
