# Supplementary material for: Distinct Organotypic Platforms Modulate Rainbow Trout (Oncorhynchus mykiss) Intestinal Cell Differentiation In Vitro
Source: Cells. 2023 Jul 13;12(14):1843. doi: 10.3390/cells12141843 (PMC10377977; doi:10.3390/cells12141843)
Supplement: Supplementary file 1 [file cells-12-01843-s001.zip › cells-2478874-supplementary.pdf]

Distinct Organotypic Platforms Modulate Rainbow Trout (*Oncorhynchus mykiss*) Intestinal Cell Differentiation In Vitro

Nicole Verdile [1,*], Federica Camin [1], Radmila Pavlovic [2,3], Rolando Pasquariello [1], Milda Stuknytė [4], Ivano De Noni [5], Tiziana A. L. Brevini [2] and Fulvio Gandolfi [1,*]

[1] Department of Agricultural and Environmental Sciences, University of Milan, 20133 Milan, Italy; federica.camin@unimi.it (F.C.)
[2] Department of Veterinary Medicine and Animal Sciences, University of Milan, 26900 Lodi, Italy; radmila.pavlovic1@unimi.it (R.P.); tiziana.brevini@unimi.it (T.A.L.B.)
[3] Proteomics and Metabolomics Facility, IRCCS San Raffaele Scientific Institute, 20132 Milan, Italy
[4] Unitech COSPECT—University Technological Platform, University of Milan, 20133 Milan, Italy; milda.stuknyte@unimi.it
[5] Department of Food, Environmental and Nutritional Sciences, University of Milan, 20133 Milan, Italy; ivano.denoni@unimi.it
* Correspondence: nicole.verdile@unimi.it (N.V.); fulvio.gandolfi@unimi.it (F.G.)

Determination of Glucose-D2 and Proline-D3 by UPLC-ESI-HR-MS

Ten µL of sample (diluted 10 : 90 with eluent B) were separated with an Acquity UPLC (Waters, Milford, MA, USA) on an Accucore 150 Amide HILIC column (150×2.1 mm, 2.6 µm) (Thermo Fisher Scientific, San Jose, CA, USA) kept at 30 °C. Eluents were 90/10 (v/v) water/200 mM aqueous ammonium formate at pH 2.8 (eluent A) and 90/10 (v/v) acetonitrile/200 mM aqueous ammonium formate at pH 2.8 (eluent B). Mobile phases were prepared as described by Park et al. (2019). For the UPLC separation, a linear elution gradient was applied (100%-to-85% of eluent B in 8 min) at a flow rate of 0.4 mL/min, followed by a column wash and re-equilibration (run-to-run time 20 min).
The LC eluate was analyzed by HR-MS on a Q Exactive instrument (Thermo Fisher Scientific) interfaced through a HESI-II probe for electrospray ionization (Thermo Fisher Scientific). The ion source and interface conditions were: spray voltage +2500 V (positive polarity) and –2000 V (negative polarity); probe heater temperature 280°C; ion transfer tube temperature 350°C; S-lens RF level 50; sheath gas pressure 35 psig; auxiliary gas pressure 15 psig. Mass spectra were acquired over the m/z range 50–500 in positive and negative polarities in a Full MS mode. The resolution was set at 70,000. The automatic gain control (AGC) target was 3×10^6, and maximum ion injection time was 200 ms.
Identification and quantification of Glc-D2 and Pro-D3 was conducted using the Xcalibur software (v3.0, Thermo Fisher Scientific) and the Glc-D2 and Pro-D3 as external standards (5 points calibration curve). Peak areas were calculated from extracted ion chromatograms of Glc-D2 and Pro-D3 deprotonated and protonated ions, $[M-H]^-$ = 181.0687 for Glc-D2 and $[M+H]^+$ = 119.0894, respectively, with 3 ppm mass tolerance. Extracted ion chromatogram of the selected sample is presented in **Figure S1**. Analyses were performed in triplicate and the results were expressed as means ± standard deviations.

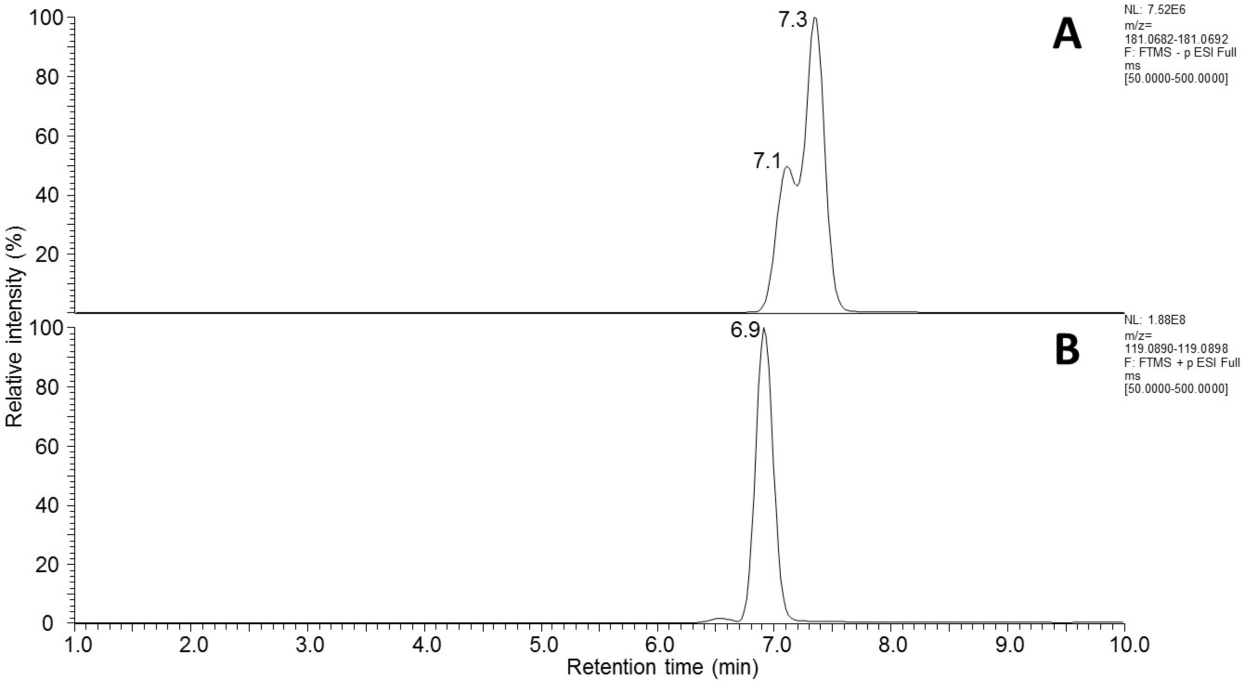

Figure S1. Extracted ion chromatograms of Glc-D2 (A) and Pro-D3 (B) of selected sample.

Bibliography

Park, S. H., Lovejoy, K., Grosse, S., De Pra, M., Meding, S., Steiner, F. (2019). Underivatized amino acid analysis in wine by HILIC separation and mass detection. Thermo Scientific Application Note 73151 (available at: https://appslab.thermofisher.com/App/4326/underivatized-amino-acid-analysis-wine-by-hilic-separation-mass-detection).
